# MicroRNA-638 inhibits cell proliferation, invasion and regulates cell cycle by targeting tetraspanin 1 in human colorectal carcinoma

**DOI:** 10.18632/oncotarget.2499

**Published:** 2014-10-07

**Authors:** Jiwei Zhang, Bojian Fei, Qifeng Wang, Mingxu Song, Yuan Yin, Binbin Zhang, Shujuan Ni, Weijie Guo, Zehua Bian, Chao Quan, Zhihui Liu, Yugang Wang, Jian Yu, Xiang Du, Dong Hua, Zhaohui Huang

**Affiliations:** ^1^ Wuxi Oncology Institute, the Affiliated Hospital of Jiangnan University, Wuxi, Jiangsu, 214062, China; ^2^ Department of Surgical Oncology, the Affiliated Hospital of Jiangnan University, Wuxi, Jiangsu, 214062, China; ^3^ Department of Pathology, Fudan University Shanghai Cancer Center, Shanghai, 200032, China; ^4^ Department of Oncology, Shanghai Medical College, Fudan University, Shanghai, 200032, China; ^5^ Department of Urology, University of Michigan Comprehensive Cancer Center, Ann Arbor, MI 48109-5942, USA; ^6^ State Key Laboratory of Oncogenes and Related Genes, Shanghai Cancer Institute, Shanghai Jiao Tong University School of Medicine, Shanghai, 200032, China

**Keywords:** microRNA-638, TSPAN1, cell proliferation, colorectal carcinoma, cell cycle

## Abstract

The expression of miR-638 was found downregulated in colorectal carcinoma (CRC) in our previous study. However, the role of miR-638 in CRC remains unknown. The aim of this study was to determine the function and mechanism of miR-638 in CRC. Here, we verified that miR-638 was frequently downregulated in CRC tissues compared with corresponding noncancerous tissues (NCTs) in an expanded CRC cohort, and survival analysis showed that the downregulation of miR-638 in CRC was associated with poor prognoses. The ectopic expression of miR-638 inhibited CRC cell proliferation, invasion and arrest the cell cycle in G1 phase, whereas the repression of miR-638 significantly promoted CRC cell growth, invasion and cell cycle G1/S transition. Subsequent mechanism analyses revealed that miR-638 inhibited CRC cell growth, invasion and cell cycle progression by targeting TSPAN1. TSPAN1 protein levels were upregulated in CRC samples and were inversely correlated with miR-638 levels. More importantly, high TSPAN1 expression levels in CRC tissues predicted poor overall survival, and appears to be an independent prognostic factor for CRC survival. Furthermore, CpG island methylation analyses revealed that the miR-638 promoter was hypermethylated in CRC and that attenuating promoter methylation was sufficient to restore miR-638 expression in CRC cells. Taken together, our current data demonstrate that miR-638 functions as a tumor suppressor in human CRC by inhibiting TSPAN1, and that TSPAN1 is a potential prognostic factor for CRC.

## INTRODUCTION

MicroRNAs (miRNAs), a class of small non-coding RNAs, play a pivotal role in many biological processes, ranging from development and differentiation to apoptosis and proliferation, by downregulating endogenous genes. In general, miRNAs exert their regulatory role on protein-coding gene expression by binding to either full or partial complementary sequences primarily in the 3′untranslated region (3′UTR) of target mRNAs [1]. Recent data show that miRNAs critically regulate tumorigenesis and progression by targeting oncogenes, tumor suppressors, or genes related to proliferation, angiogenesis, and apoptosis [2].

Different tumor types and tumors at various differentiation stages exhibit unique miRNA profiles, suggesting important and complex regulation effects of miRNA in tumorigenesis [3]. Recent advances have resulted in the promising application of miRNAs as potential biomarkers for cancer diagnostics, progression, and response to treatment [3–7]. Although, many papers have reported that miRNAs are involved in human cancers, the biological functions and molecular mechanisms of many miRNAs remain largely unknown.

Colorectal carcinoma (CRC) is one of the most common cancers. Although the prognosis has improved slightly in the past years, CRC remains the third commonest cancer and the third leading cause of cancer death among men and women worldwide [8, 9]. In the past decade, many miRNAs were identified as associated with CRC development, including miR-17 [10], miR-34 [11], miR-93 [12], miR-137 [13], miR-143 [14], and miR-145 [15]. Our previous work revealed that miR-95, which is overexpressed in CRC, could promote CRC cell proliferation by targeting SNX1 [16]. In addition, we and other groups also found that miRNAs appear to be potential biomarkers for the early detection of CRC [4–6, 17, 18]. All of these studies provide a strong theoretical basis for the key role of miRNA in CRC tumorigenesis, and highlight the promising implication of miRNA in the diagnosis and prognosis of CRC.

Our previous expression profiling data revealed that miR-638 was downregulated in CRC compared with the corresponding noncancerous tissues (NCTs) [16]. Recently, the downregulation of miR-638 has also been reported in gastric cancer [19, 20], lung cancer [21], and CRC [22], suggesting that miR-638 might play an important role in human tumorigenesis. However, little was known about the exact function and potential mechanisms of miR-638 in human tumors [21–23]. In the present study, we validated that the miR-638 expression was significantly decreased in CRC compared with NCT in an expanded CRC cohort, and miR-638 can inhibit CRC cell proliferation, invasion and induce cell cycle arrest in G1 phase by directly targeting TSPAN1, a molecular that appears to be an independent prognostic factor for CRC. In addition, we revealed that promoter methylation appear to be an important mechanism medicating the silencing of miR-638 in CRC.

## RESULTS

### Downregulation of miR-638 in CRC predicts poor survival

**Figure 1:**
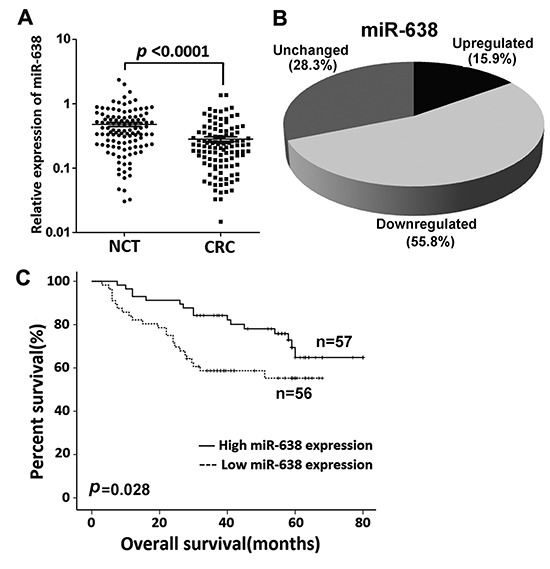
miR-638 expression is frequently reduced in CRC **(A)** miR-638 expression was detected by quantitative reverse transcription polymerase chain reaction (qRT-PCR) in 113 paired CRC and adjacent noncancerous tissues (NCTs). miR-638 expression was markedly downregulated in tumor tissues when compared to the corresponding NCTs (U6 small nuclear RNA was used as an internal control). **(B)** miR-638 expression is frequently decreased (more than 1.5-fold) in CRC (55.8%). **(C)** Overall survival analysis based on the expression level of miR-638 in CRC tissues.

The data for the miRNA expression microarray showed that miR-638 was downregulated in CRC [16]. To further confirm this result, we used qRT-PCR to examine the expression level of mature miR-638 in an expanded cohort of 113 CRC patients. The statistical results were consistent with the expression microarray data, and miR-638 expression was remarkably downregulated (more than 1. 5-fold) in 55.8% (63/113) of the CRC tissues compared to the corresponding NCTs (*p* < 0.0001, Figure 1A and B). The relative expression levels of miR-638 in 8 CRC cell lines were also much lower than in normal colon epithelium mucosae (Supplementary Figure S1). No significant relationship was found between miR-638 expression in CRC and tumor size, location, stage, or grading (*p* > 0.05), but patients with low miR-638 expression showed shortening survival when compared to patients with high miR-638 expression (*p* = 0.028, Figure 1C). To further evaluate the prognostic effect of miR-638, we performed a multivariable analysis. After adjustment for age, gender, tumor size, TNM stage and grading, a Cox multivariate analysis indicated that miR-638 expression is a potential prognostic factor for survival (adjusted HR = 0.392, 95% CI = 0.201-0.776, *p* = 0.006)

### miR-638 inhibits CRC cell proliferation, invasion and regulates cell cycle G1/S transition

**Figure 2:**
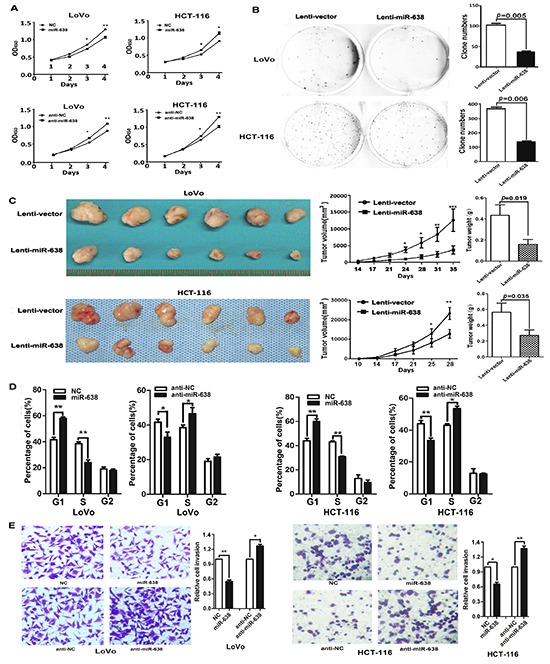
miR-638 inhibits CRC cell proliferation, invasion and regulates cell cycle progression **(A)** The upregulation of miR-638 repressed the proliferation of HCT-116 and LoVo cells, whereas the knockdown of miR-638 enhanced the cellular growth rate of HCT-116 and LoVo cells. The cell counting Kit-8 assay was used to determine the cell growth rate. * *p*<0.05, ** *p*<0.01, compared to the negative control (NC). **(B)** HCT-116 and LoVo cells stably expressing miR-638 showed decreased colony formation rates compared to the control cells. **(C)** The effect of miR-638 on tumor formation in a nude mouse xenograft model. LoVo and HCT-116 cells stably expressing miR-638 or the control (2×10^6^) were injected s.c. into the right flank of each nude mouse. The tumor volume and weight of the miR-638 group were significantly decreased compared to the control group (*p<0.05, ** *p*<0.01, *** *p*<0.001). **(D)** Cell cycle analysis of LoVo and HCT-116 cells transfected with miR-638, anti-miR-638 or NC. miR-638 induced G1-phase arrest in CRC cells (*p<0.05, ** *p*<0.01). **(E)** miR-638 overexpression repressed the invasion of LoVo and HCT-116 cells, whereas the silencing of miR-638 enhanced the invasion of LoVo and HCT-116 cells (*p<0.05, ** *p*<0.01).

The decreased expression of miR-638 in CRC suggests that miR-638 may contribute to tumorigenesis. A cell proliferation assay showed that the ectopic expression of miR-638 significantly reduced the growth of LoVo and HCT-116 cells, whereas the silencing of miR-638 significantly promoted cell proliferation (*p* < 0.01, Figure 2A). The results of a clony formation assay confirmed that the overexpression of miR-638 can repress the clony formation of CRC cells (*p* < 0.01, Figure 2B). To evaluate the *in vivo* function of miR-638, a tumor formation assay in a nude mouse model was performed using LoVo and HCT-116 cells stably expressing miR-638. The overexprssion of miR-638 significantly repressed tumorigenesis compared with the vector control (*p* < 0.05, Figure 2C). Given that miR-638 inhibited CRC cell proliferation, we next sought to exam whether miR-638 has any impact on cell cycle progression of CRC cells. As shown in Figure 2D, cell number in G1 phase was significantly elevated and the cell population in S phase reduced in miR-638-overexpressing LoVo and HCT-116 cells compared with control cells. In contrast, the cell population of G1 phase was reduced and cell number in S phase was increased in miR-638-depleted CRC cells (Figure 2E). Together, these data suggest that miR-638 inhibit CRC proliferation by repressing the cell cycle progression at the G1/S transition in CRC cells. In addition, to determine whether miR-638 could modulate the metastasis ability of CRC, we examined the effect of miR-638 on CRC cell invasion using a transwell assay. As shown in Figure 2E, miR-638-transfected CRC cells exhibited considerably slower invasion compared with the control cells, whereas the silencing of miR-638 enhanced the invasion of LoVo and HCT-116 cells (Figure 2E).

### Screening of candidate target genes of miR-638

**Figure 3:**
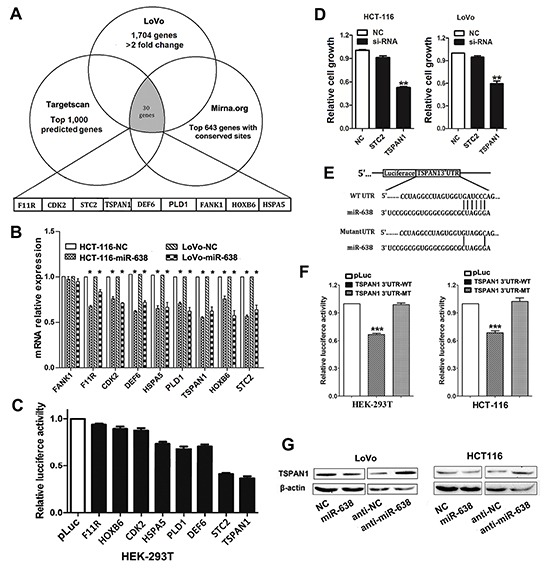
Screening of candidate target genes of miR-638 in CRC **(A)** Initial screening of miR-638 target genes in LoVo cells transfected with miR-638 mimic using microarray and bioinformatic predictions. A total of nine downregulated genes were selected from the 30 genes in the initial screening basing on the functional analysis. **(B)** Validation of the microarray results in both HCT-116 and LoVo cells by quantitative reverse transcription polymerase chain reaction (qRT-PCR). A total of eight genes were indeed downregulated in the miR-638-transfected CRC cells compared to the control cells (*p<0.05). **(C)** Two candidate genes (STC2 and TSPAN1) were selected for their significantly decreased luciferase activity in the miR-638-transfected HEK-293T cells. **(D)** Proliferation assays performed using CRC cell lines (HCT-116 and LoVo) transfected with siRNA directed against two candidate genes. Depleted TSPAN1 expression showed the most obvious growth repression effect. ** *p*<0.01 compared to NC. **(E)** Schematic of the wild-type or mutant 3′UTRs of the TSPAN1 vector constructs. The complementary site of the seed region of miR-638 was selected for mutation. **(F)** The relative luciferase activity assays of luciferase reporters with the TSPAN1 wild-type (WT) or mutant-type (MT) 3′UTR were performed in HEK-293T and HCT-116 cells after cotransfection with miR-638 mimic. Luciferase activity was determined at 24 h after transfection and normalized to the Renilla luciferase activity. The luciferase activity generated by pLuc in each experiment was set as 1(***p<0.001 compared to the pLuc control). **(G)** The protein levels of TSPAN1 were determined by western blot assays using LoVo and HCT-116 cells transfected with miR-638 mimic, miR-638 inhibitor (anti-miR-638) or the corresponding negative control (NC or anti-NC). Beta-actin protein served as an internal control.

To investigate the molecular mechanism by which miR-638 suppresses CRC cell proliferation, genomic-wide expression profiling was first performed in miR-638- or NC-transfected LoVo cells using a microarray. Compared to the control, a total of 1,704 downregulated genes (>2-fold change) were identified in the miR-638-transfected LoVo cells (Supplementary Table S1). TargetScan and miRanda algorithms were then used to search for putative protein-coding gene targets of miR-638. By comparing all of the downregulated genes with the candidate genes predicted by the programs, a total of 30 downregulated genes were selected (Figure 3A). Because it is generally accepted that miRNAs exert their function by inhibiting the expression of their target genes, miR-638 may execute its tumor-inhibiting function by downregulating targets that normally have tumor-promoting function. Based on this rationale, 9 candidate genes (CDK2, DEF6, FANK1, F11R, HOXB6, HSPA5, PLD1, STC2, and TSPAN1) were selected from the 30 genes. We used qRT-PCR to verify the 9 candidate genes in HCT-116 and LoVo cells transfected with miR-638, and found that 8 of the 9 genes were downregulated in the miR-638-transfected cells compared with the control cells (Figure 3B).

The 3′UTRs of these 8 genes containing predicted binding sites of miR-638 were cloned into a luciferase reporter vector to evaluate the influence of miR-638 on the expression of a reporter gene using a luciferase assay. As showed in Figure 3C, the expression of the reporter gene in the recombinant plasmids containing the 3′UTR of TSPAN1, STC2, PLD1, DEF6 or HSPA5, were significantly inhibited by miR-638, particularly STC2 and TSPAN1 (Figure 3C). Therefore, we chose TSPAN1 and STC2 as candidate targets of miR-638 for the subsequent functional analysis. A cell proliferation assay was performed on HCT-116 and LoVo cells transfected with TSPAN1- or STC2-specific siRNAs (Supplementary Figure S2). In both HCT-116 and LoVo cells, the siRNA- mediated silencing of TSPAN1 resulted in decreased cell proliferation, whereas no significant growth-inhibiting effect was observed in STC2-depleted CRC cells (Figure 3D). Taken together, these data suggest that TSPAN1 is a potential functional target of miR-638 in CRC.

### miR-638 posttranscriptionally reduces TSPAN1 expression by targeting its 3′UTR

To further confirm that TSPAN1 is a direct target of miR-638 in CRC, we mutated the predicted binding site of miR-638 in the TSPAN1 3′UTR (Figure 3E), and found that the mutant 3′UTR was completely refractory to miR-638-mediated luciferase reporter repression in both HEK-293T and HCT-116 cells (Figure 3F). In agreement with these results, the endogenous TSPAN1 protein levels were also decreased in miR-638-overexpressing CRC cells and could be restored in miR-638-depleted CRC cells (Figure 3G).

### TSPAN1 levels are negatively correlated with miR-638 expression and CRC survival

**Figure 4:**
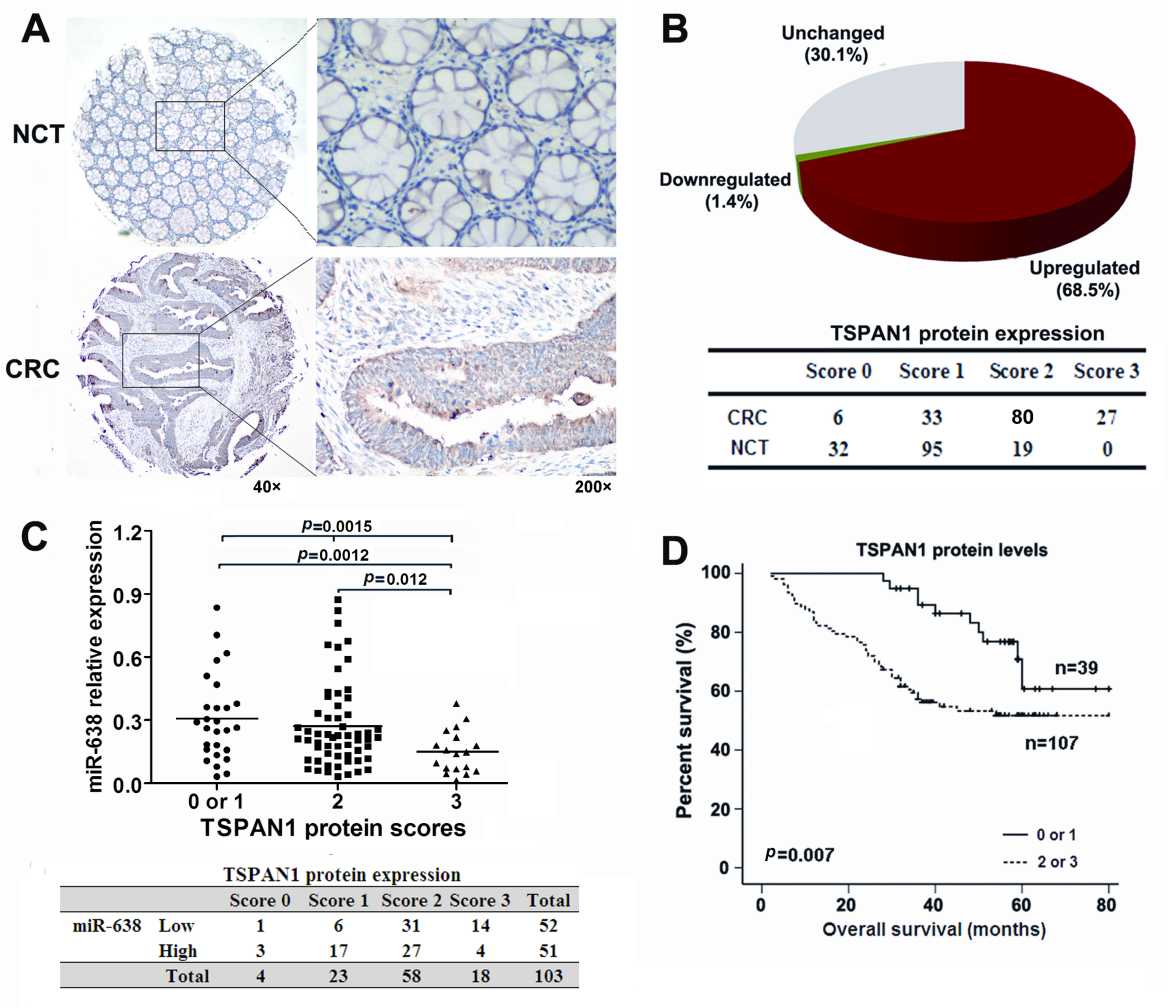
TSPAN1 is overexpressed in CRC and its levels are inversely correlated with the levels of miR-638 **(A)** Immunohistochemical staining of TSPAN1 in 146 tumor tissues and the adjacent noncancerous tissues (NCTs). Brown cytoplasmic TSPAN1 staining was observed in CRC tissues but nearly absent in the normal epithelia. **(B)** TSPAN1 protein expression was frequently increased in the tumor tissues compared to the matched NCTs. (**C)** The expression levels of TSPAN1 negatively correlated with the miR-638 levels in CRC tissues (Spearman *r* =−0.341, *p* <0.0001; Chi-Square=12.084, *p* = 0.005). (**D)** Overall survival analysis based on the expression levels of TSPAN1. The groups were ranked according to the TSPAN1 staining intensity. The percent of overall survival in patients with low TSPAN1 expression (scored 0 or 1) was significantly higher than that of the patients with high TSPAN1 expression (scored 2 or 3) (Log-rank=7.272, *p* =0.007).

To further evaluate the relationship between TSPAN1 and miR-638 in human CRC, we measured the TSPAN1 protein expression in 146 paired CRC and NCT samples using immunohistochemistry methods (Supplementary Figure S3). Of the 146 cases, 100 tumors showed increased TSPAN1 expression compared to the paired NCTs (Figure 4A and B). Both miR-638 and TSPAN1 expression data were available for a total of 103 paired tumors and NCTs, and the results showed that the protein levels of TAPAN1 in the CRC tissues were inversely correlated with the miR-638 levels (Spearman *r* = −0.341, *p* < 0.0001; Figure 4C), suggesting that the increased TSPAN1 expression in CRC might due to miR-638 underexpression. In addition, increased TSPAN1 immunoreactivity correlated with poor overall survival (Log-rank = 7.272, *p* = 0.007, Figure 4D), poor tumor differentiation (Spearman *r* = 0.184, *p* = 0.026) and high TNM stage (Spearman *r* = 0.170, *p* = 0.040). After adjustment for age, gender, tumor size, TNM stage and grading, a Cox multivariate analysis indicated that high TSPAN1 expression is an independent risk factor for survival (adjusted HR = 2.818, 95% CI = 1.355-5.862, *p* = 0.006) (Table 1).

**Table 1:** Cox multivariate analyses for overall survival

Variables	Overall survival		*P* value
	HR	95%CI	
Age (≥58 *vs*.<58)	1.071	0.618–1.856	0.807
Gender (male *vs*. female)	0.636	0.364–1.111	0.112
Tumor size(<5 *vs*.≥5cm)	1.317	0.775–2.238	0.309
Grading (G1-2 *vs*.G3)	1.739	0.994–3.204	0.076
TNM stage			
I	1.0 (referent)		
II	3.265	0.407–26.225	0.266
III	13.114	1.782–96.514	0.011
IV	60.303	7.782–461.924	<0.0001
TSPAN1 protein levels (0-1 vs. 2-3)	2.818	1.355–5.862	0.006

HR: hazard ratio; CI: confidence interval; TNM: tumor–node–metastasis classifications.

### miR-638 represses cell proliferation, invasion and regulates cell cycle progression by targeting TSPAN1 in CRC

**Figure 5:**
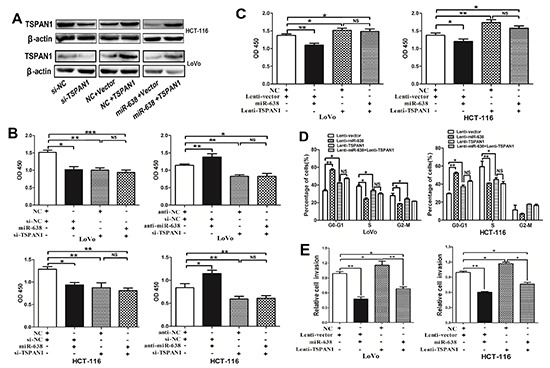
miR-638 represses CRC cell growth, invasion and cell cycle progression by downregulating TSPAN1 expression **(A)** TSPAN1 protein expression was determined in CRC cells using Western blotting. **(B)** The knockdown of TSPAN1 by siRNA significantly repressed CRC cell proliferation, and upregulation of miR-638 did not suppress cell growth in TSPAN1-depleted HCT-116 or LoVo cells. miR-638 silencing promoted cell growth, but did not promote cell proliferation in TSPAN1-depleted CRC cells. **(C)** The upregulation of TSPAN1 (ORF without 3′UTR) markedly promoted cell growth and abrogated miR-638-induced cell growth inhibition in HCT-116 or LoVo cells. **(D)** Cell cycle analyses were performed in LoVo and HCT-116 cells transfected with miR-638, TSPAN1 or vector control (*p < 0.05; **p < 0.01; ***p < 0.001; NS, not significant). (E) Upregulation of the TSPAN1 promoted cell invasion and abrogated miR-638 induced invasion-inhibition in CRC cells (*p < 0.05; **p < 0.01).

To further evaluate whether TSPAN1 is a direct functional target of miR-638 in CRC cells, we performed a series of functional restoration assays using HCT-116 and LoVo cells. We inhibited TSPAN1 expression with siRNA and found that the TSPAN1-depleted cells exhibited decreased cell proliferation, which phenocopied the proliferation-repressing effect of miR-638, whereas miR-638 silencing could not restore cell proliferation in TSPAN1-depleted CRC cells (Figure 5A and B). Furthermore, the ectopic expression of TSPAN1 using a plasmid of TAPAN1 open reading frame (ORF) promoted cell proliferation, which could not be repressed by the overexpression of miR-638 (Figure 5A and C). Likewise, TSPAN1 overexpression could significantly abrogate miR-638-dependent inhibitory effect on the G1/S transition and cell invasion (Figure 5D and 5E, Supplementary Figure S4). Collectively, these data prove that miR-638 represses CRC cell proliferation, invasion and cell cycle progression by directly targeting TSPAN1.

### Downregulation of miR-638 is caused by promoter hypermethylation in CRC cells

**Figure 6:**
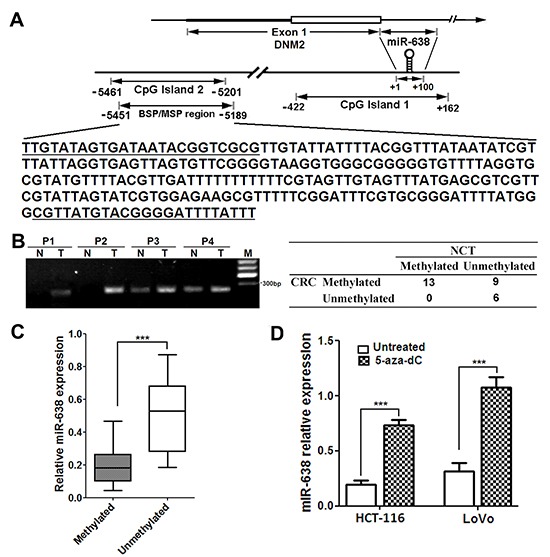
The promoter CpG island of miR-638 gene is hypermethylation in CRC cells (**A**) Schematic illustration of the spatial arrangement of CpG islands in the promoter region of miR-638 gene and the selected target sequences used for the DNA methylation analysis. The underlined sequences are regions corresponding to the primers used. (**B**) The results of methylation-specific PCR analysis in 28 paired CRC tumors and noncancerous tissues (NCTs). P1-4, CRC patients; N, NCT; T, tumor; M, DNA marker. **(C)** Methylated tumors showed decreased miR-638 expression compared with unmethylated tumors(*** *p* < 0.001). (**D**) Quantitative analysis of miR-638 expression in HCT-116 and LoVo cells treated with 10μM 5-aza-dC for 72 hours. *** *p* < 0.001 compared to the untreated control.

To investigate the possible molecular mechanisms that mediate the downregulation of miR-638 in CRC, we performed a promoter methylation analysis. Two CpG islands were found in the promoter region of miR-638 gene using online database tools (http://genome.ucsc.edu/cgi-bin/hgGateway) (Figure 6A). The first CpG island overlaps with the miR-638 sequence and the second CpG island is located in ~5Kb upstream of the miR-638 gene. The results showed that the methylation ratio at the second CpG island in CRC tissues (22/28, 78.6%) was significantly higher than that in NCTs (14/28, 50.0%) (*p* = 0.026) (Figure 6B), and all five normal colonic epithelia samples were unmethylated. Furthermore, methylated tumors showed decreased miR-638 expression compared with unmethylated tumors (Figure 6C). Subsequent BSP analysis confirmed the hypermethylation at the CpG island in CRC cell lines and clinical CRC tumors (Supplementary Figure S5A and B). In contrast, few samples were hypermethylated in the first CpG island (4/28, 14.3% in both tumors and NCTs)(data not shown). To further confirm the effect of promoter hypermethylation on the expression of miR-638, HCT-116 and LoVo cells were treated with the DNA methyltransferase inhibitor 5-aza-dC, and we found that the expression of miR-638 in the treated CRC cells was significantly restored compared with untreated cells (Figure 6E). Taken together, these data suggest that miR-638 downregulation in CRC cells is attributable to promoter methylation.

## DISCUSSION

Recent investigations have shown that the dysregulation of miRNAs is a common event in human tumorigenesis, and miRNAs appears to be promising tumor biomarkers [3, 4, 24]. The effects of miRNAs in tumorignensis are based on their posttranscriptional regulation of the expression of many cancer-related genes. As we previously reported, many miRNAs are aberrantly expressed in CRC, suggesting that variations of miRNA expression are common events in colorectal tumorigenesis [16, 25]. In addition, miRNAs present a novel prognostic and diagnostic tool in patients with a high risk of CRC or with a CRC diagnosis [4–6, 17].

To identify new miRNAs associated with CRC, we performed miRNA expression profiling and found that miR-638 is significantly downregulated in CRC, suggesting that miR-638 is a new CRC-associated miRNA [16, 22]. Interestingly, miR-638 has also been reported to be aberrantly expressed in other human tumors [19–22]. Xia et al showed that the downregulation of miR-638 promotes proliferation and invasion by regulating SOX2 and induces epithelial-to-mesenchymal transition (EMT) in non-small-cell lung cancer [21]. A recent paper showed that miR-638 could inhibit cell growth by targeting Sp2 in gastric cancer [23]. These data show the extensive function of miR-638 in tumorigenesis. However, the exact function and mechanisms of miR-638 in CRC are largely unknown. In this study, we confirmed the downregulation of miR-638 in CRC tissue in an expanded cohort of CRC, and revealed the favourable effect of miR-638 on the survival of CRC patients. Functional studies provide the first line of evidence that miR-638 can inhibit cell growth and cell cycle progression at the G1/S transition in CRC cells. In addition, we also observed the inhibitory effect of miR-638 on CRC cell invasion.

Tetraspanins is a large family of ubiquitously expressed membrane proteins. Several tetraspanins molecules, such as CD9, CD82, CD63 and CD151, are implicated in the regulation of cell differentiation, proliferation, and motility [26]. TSPAN1, a new member of the tetraspanin family, is a recently discovered tumor-related gene [27–29]. TSPAN1 was found to be overexpressed in human CRC [27, 29], hepatocellular carcinoma [30], gastric cancer [31], and ovarian carcinomas [32]. In the present study, we revealed, for the first time, that TSPAN1 is a functional target of miR-638 in CRC. Further functional and clinical analyses showed that the suppression of TSPAN1 inhibits cell proliferation and invasion in CRC and that high TSPAN1 protein expression appears to be an independent prognostic factor for CRC, consistent with the results of other groups [27, 29], suggesting the key role of TSPAN1 in CRC. Our data demonstrated that the overexpression of TSPAN1 is due to the underexpression of miR-638 in CRC. The detailed mechanism by which TSPAN1 promotes CRC cell proliferation and invasion should be investigated in future work. Interestingly, a recent study reported that the downregulation miR-638 can promote CRC cell invasion and EMT though inhibiting SOX2, a target of miR-638 reported in lung cancer [21, 22], confirming that miR-638 exerts important function in CRC by regulation different targets.

CpG island promoter hypermethylation has been shown to trigger the inactivation of tumor suppressor genes, including miRNAs [33, 34]. The miR-638 gene is an intronic miRNA located on the first intron of DNM2. We found that one CpG island located ~5-kb upstream of the miR-638 sequence is hypermethylated in most of CRC tissues and cell lines. Interestingly, we also found moderate or weak levels of methylation in about half of the corresponding NCTs. In contrast, no significant methylation was found in normal colonic epithelia samples. A plausible explanation is that morphological normal NCTs are often not really normal and some aberrant molecular changes occur in these tissues, and the hypermethylation of the CpG island is likely an early event in CRC carcinogenesis. Quantitative methylation analysis should be performed in CRC tissues to filter out low level background methylation by setting a proper cutoff value and therefore could determine the exact relationship between the promoter methylation levels and the miR-638 expression. In addition, attenuating promoter methylation was sufficient to restore miR-638 expression in CRC cells. A recent study [35] showed that DNM2 along with miR-99a (that derived from the opposite strand of DNM2) reciprocally regulate HIFs and ovarian cancer metastasis, suggesting a tumor-suppressive role of DNM2. We revealed that DNM2 expression was frequently downregulated in CRC and positively correlated with miR-638 expression, indicating that they may share common regulatory mechanisms (Supplementary Figure S6A and B). The downregulation of DNM2 in CRC was further confirmed in a data available in GEO database (GSE41258) (Supplementary Figure S6C). Taken together, our primary data indicate that promoter hypermethylation is a potential mechanism for miR-638 underexpression in CRC. The potential regulation of DNA methylation on miR-638/DNM2 should be elucidated in future work.

Previous studies show that copy number variants (CNVs) also result in the aberrant expression of human genes, including miRNAs [25]. We analyzed CNVs in 44 paired significant changes CRC and adjacent NCT tissues and didn't observed with regard to the copy numbers of miR-638 gene between CRC and NCT samples (data not shown), suggesting that CNV may not be an important mechanism leading to the downregulation of miR-638. The analysis of more clinical samples is necessary to confirm these findings.

In summary, we determined that miR-638 is frequently downregulated in CRC, which is attributed to, at least in part, the promoter methylation. miR-638 inhibited CRC proliferation, invasion and cell cycle progression by sliencing TSPAN1, a molecule that is closely related to the prognosis of CRC.

## MATERIALS AND METHODS

### Cell lines and cell cultures

Eight human CRC cell lines, including Caco2, DLD1, HCT8, HCT-116, HT29, LoVo, SW480, and SW620, were purchased from American Type Culture Collection (ATCC). DLD1, HT29, Caco2 and HEK-293T cells were cultured in DMEM. HCT-116, HCT-8, and LoVo cells were cultured in McCoy's 5a, RPMI-1640, and F12-K medium, respectively. SW480 and SW620 cells were maintained in Leibovitz's L-15 medium. All of the media (Hyclone, USA) were supplemented with 10% fetal bovine serum (Gibco, USA). The cells were incubated under the conditions recommended by ATCC.

### Human tissues

A total of 156 pairs of primary CRC tissues and their adjacent NCTs were collected from 2005 to 2008 at the Affiliated Hospital of Jiangnan University and Fudan University Shanghai Cancer Center. Of the 156 cases, 146 were subjected to the TSPAN1 detection and 113 were examined for their miR-638 expression. All of the human material was obtained with informed consent, and the study was approved by the clinical research ethics committee of Fudan University Cancer Center and the Affiliated Hospital of Jiangnan University. The detailed clinical information is included in supplementary Table S2.

### DNA and RNA extraction and quantitative real-time RT-PCR

Tissue genomic DNA was isolated using the General AllgGen Kit (Cwbio, China) according to the manufacturer's protocol. All RNA was purified using the TRIzol reagent (Invitrogen, USA). The concentrations of DNA and RNA were determined using a NanoDrop ND-1000 instrument (NanoDrop, USA), and aliquots of the samples were stored at −80°C. The relative DNA copy numbers were determined by qPCR using SYBR Premix Ex Taq (TaKaRa, Japan) with 50 ng DNA as the template in a final volume of 20 μL and LINE1 was used as an internal control[36, 37]. cDNA was synthesized using the PrimeScript RT reagent kit (TaKaRa, Japan). qRT-PCR analyses were performed to quantitate mRNA relative expression using SYBR Premix Ex Taq with beta-actin as an internal control. TaqMan microRNA assays (Applied Biosystems, USA) were used to determine the expression levels of miR-638 with U6 small nuclear RNA as an internal control. The qRT-PCR results were defined based on the threshold cycle (Ct), and the relative expression levels were calculated using the 2^−ΔΔCt^ method [38]. PCR was performed using an ABI Vii7 instrument (Applied Biosystems, USA).

### Oligonucleotide transfection

An siRNA sequence for TSPAN1 (5′-AGUGCCUGCCAUCAAGAAATT-3′), miR-638 mimics and inhibitor (anti-miR-638, chemically modified antisense oligonucleotides designed to specifically target mature miR-638) were synthesised by Ribobio (China). Oligonucleotide transfection was performed using Lipofectamine 2000 reagent (Invitrogen, USA) according to the manufacturer's instructions. The final concentration of the miR-638 mimics or inhibitors in the transfection mixture was 50 nM.

### Vector constructs

The sequence of human pri-miR-638 was amplified from normal human genomic DNA by nested PCR using PrimerSTAR Premix (TaKaRa, Japan). The sequence was then cloned into the lentivirus expression vector pWPXL to generate pWPXL-miR-638. The predicted binding sites in the 3′UTRs of the potential target genes of miR-638 were amplified by nested PCR and cloned into the region directly downstream of a CMV promoter-driven firefly luciferase cassette in the pcDNA3.0 vector (pLuc) [16]. The mutant 3′UTR of TSPAN1, which carries the mutated sequence in the complementary site of the seed region of miR-638, was generated from the pLuc-TSPAN1 3′UTR-WT plasmid by overlap-extension PCR. DNA sequencing was used to confirm all of these vector constructs. The ORF of TSPAN1 was amplified via nested PCR and cloned into the pWPXL vector. The primers used for the vector constructs are shown in supplementaryTable S3.

### Lentivirus production and transduction

Plasmids of pWPXL, pWPXL-miR-638 or pWPXL-TSPAN1 were transfected into HEK-293T cells using Lipofectamine 2000 reagent (Invitrogen, USA) with the packaging plasmid ps-PAX2 and the envelope plasmid pMD2G; the virus particles were harvested 48 hours later. HCT-116 and LoVo cells were infected with recombinant lentivirus-transducing units plus 6 μg/mL polybrene (Sigma, USA). The resulting stable cell lines were validated using qRT-PCR and/or Western blotting.

### Cell proliferation assay and colony formation assay

Cell proliferation ability was detected using the Cell Counting Kit-8 (CCK8; Dojindo Laboratories, Japan) according to the manufacturer's instructions. Colony formation assays were performed as previously described [16].

### Cell invasion assay

The invasion ability of CRC cells was tested in a Transwell Boyden Chamber (8-μm pore size, BD Biosciences, USA) as previously described [25].

### Cell cycle analysis

Cells were collected and fixed in ice-cold 70% ethanol overnight. The fixed cells were washed with phosphate-buffered saline and subjected to the cell cycle analysis using the Cell cycle Detection Kit (KeyGEN, China) according to the manufacturer's instructions.

### Tumor formation assay in a nude mouse model

Athymic male BALB/c nude mice were maintained under specific pathogen-free conditions in the Experimental Animal Department of Fudan University. LoVo or HCT-116 cells stably expressing miR-638 or the vector control were washed from subconfluent cell culture plates with PBS and then resuspended with DMEM at a concentration of 1×10^7^ cells/mL. An aliquot (0.2 mL) of suspended LoVo or HCT-116 cells was subcutaneously injected into the right flank of each mouse at the age of 5 weeks (n=8 for each groups). After transplantation, the growth of the subcutaneous tumors was assessed twice a week. The tumor size was monitored as previous described [16]. The mice were sacrificed after a period of 4–6 weeks. The protocols of the animal model were approved by the Shanghai Medical Experimental Animal Care Commission.

### Microarray analysis

Expression profiling was performed using an Agilent Human Whole Genome Oligo Microarray (4×44K) that contains more than 42,034 genes and transcripts (Agilent, USA). A total of 5×10^5^ LoVo cells were seeded in 6 cm^2^ culture plates and transfected with miR-638 mimic or negative control as described above. After propagation for 48 hours, total RNA was extracted as described above. The RNA concentration was determined, and the integrity of the RNA was verified using an Agilent 2100 bioanalyzer (Agilent, USA). The microarray profiling was performed as described in our previous work [25]. An mRNA was designated as “downregulated” if its expression in miR-638-transfected cells was greater than 2-fold lower than that in the corresponding control cells.

### Luciferase assay

Approximately 5,000 HEK-293T cells per well and 12,000 HCT-116 cells per well were plated into 96-well plates and were cotransfected with 50 nM of miR-638 mimic (or NC), 50 ng of the luciferase reporter, and 5 ng of the pRL-CMV Renilla luciferase reporter using 0.5 μL Lipofectamine 2000 (Invitrogen, USA) per well. After 48-hours of incubation, the firefly and Renilla luciferase activities were quantified using a dual-luciferase reporter assay (Promega, USA).

### Western blotting

Total protein was first separated by 12% sodium dodecyl sulphate-polyacrylamide gel electrophoresis and then transferred to nitrocellulose membranes (Millipore, USA). The membranes were blocked with 5% nonfat milk and incubated with a rabbit anti-TSPAN1 polyclonal antibody at a dilution of 1:1000 (Abcam, USA) or a mouse anti-beta-actin monoclonal antibody at a dilution of 1:2,000 (Sigma, USA). The membranes were subsequently incubated with a goat anti-mouse or anti-rabbit horseradish peroxidase secondary antibody (Sigma, USA). The protein complex was detected using enhanced chemiluminescence reagents (Pierce, France). Beta-actin was used as an internal control.

### Immunohistochemical staining

Tissue arrays were constructed using 146 paired CRC tissues and NCTs. Immunohistochemical staining was performed on 4 μm sections of paraffin-embedded tissues to detect the expression levels of the TSPAN1 protein. In brief, the slides were incubated with anti-TSPAN1 antibody (Abcam, USA) diluted 1:200 overnight at 4°C. The subsequent steps were performed using the EnVision™ FLEX High pH 9.0 visualization system according to the manufacturer's protocols (DAKO, Demark). The TSPAN1 staining intensity measurements are presented in supplementary Figure S3.

### Promoter methylation analysis

Genomic DNA from CRC cell lines, CRCs and adjacent NCTs was bisulfite-modified as previously described [39]. The Bisulfite-treated DNA was amplified using bisulfite-sequencing PCR (BSP) primers located in the promoter region of miR-638. The purified BSP products were directly sequenced and the methylation status of each CpG site was determined as described previously [40]. The PCR products were cloned using the pMD18-T Vector System (Takara, Japan). Plasmids from single colonies were purified and sequenced. To perform the methylation-specific PCR (MSP) analysis, the bisulfite-treated DNA from the CRC clinical samples was subjected to PCR, and the PCR products were analyzed by 2% agarose gel electrophoresis. The BSP and MSP primers were designed using MethPrimer (Supplementary Table S3) [41]. HCT-116 or LoVo cells were plated in a 25-cm^2^ flask. Twenty-four hours later, cells were treated with 10 μM demethylating agent, 5-aza-dC (Sigma, USA); the medium was changed at 72 h after treatment. RNA was extracted from the treated cells using the TRIzol reagent and was subjected to a qRT-PCR analysis for miR-638.

### Statistical analyses

The results are presented as the mean values ± SEM. The data were subjected to Student's *t*-tests unless otherwise specified (χ2 test, Spearman's correlation). The survival curves were plotted according to the Kaplan-Meier method, with the log-rank test applied for comparisons. Cox proportional hazard regression analysis was used to estimate the Hazard Ratios (HRs) and their 95% confidence intervals (CIs). A *p* value of less than 0.05 was considered statistically significant. SPSS 16.0 package (IBM, USA) and Graphpad prism 5.0 software (GraphPad Software, USA) were used for the statistical analyses and scientific graphing, respectively.

## SUPPLEMENTARY FIGURES AND TABLES






