# Supplementary material for: MicroRNA-638 inhibits cell proliferation, invasion and regulates cell cycle by targeting tetraspanin 1 in human colorectal carcinoma
Source: Oncotarget. 2014 Oct 7;5(23):12083–96. doi: 10.18632/oncotarget.2499 (PMC4322991; doi:10.18632/oncotarget.2499)
Supplement: Supplementary file 2 [file oncotarget-05-12083-s002.pdf]

SUPPLEMENTARY FIGURES AND TABLES

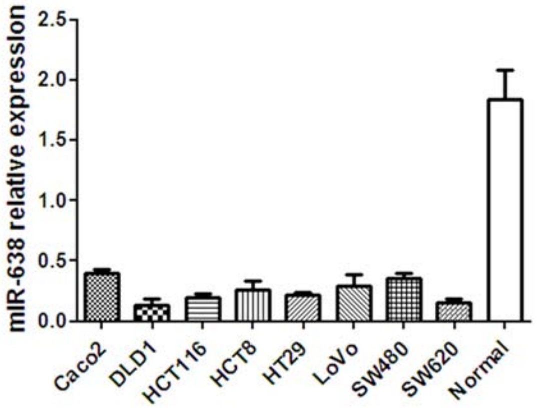

Supplementary Figure S1: The expression of miR-638 in CRC cell lines. Five samples of normal colon epithelium mucosae were pooled and subjected to quantitation of miR-638 expression (Normal).

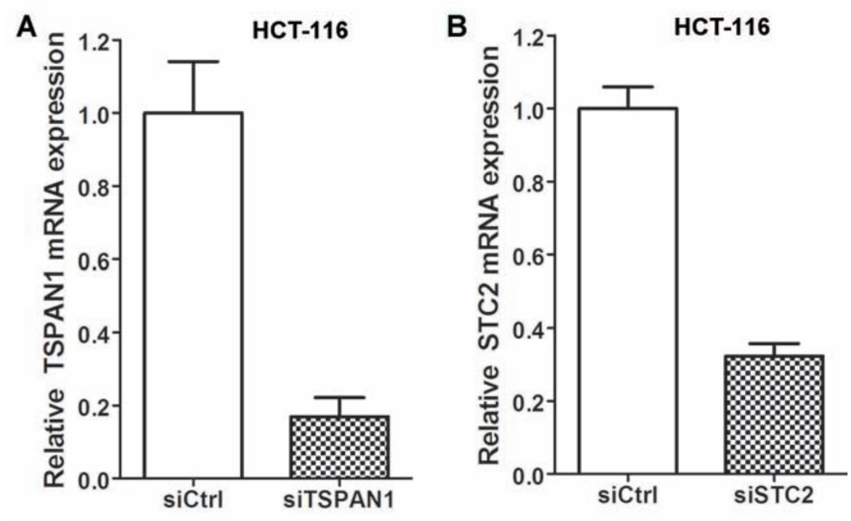

Supplementary Figure S2: Knockdown of TSPAN1 (A) and STC2 (B) using TSPAN1- or STC2- specific siRNAs in HCT-116 cells.

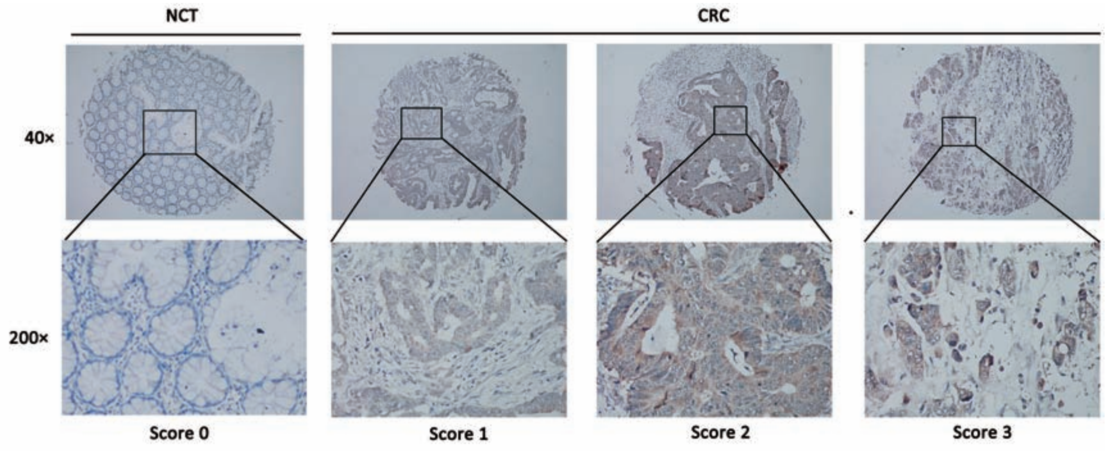

Supplementary Figure S3: Immunohistochemical staining of TSPAN1 in the CRC and NCT samples. TSPAN1 protein is mainly localized in the cytoplasm. The scores (0, 1, 2 and 3) are based on the intensity of the brown staining.

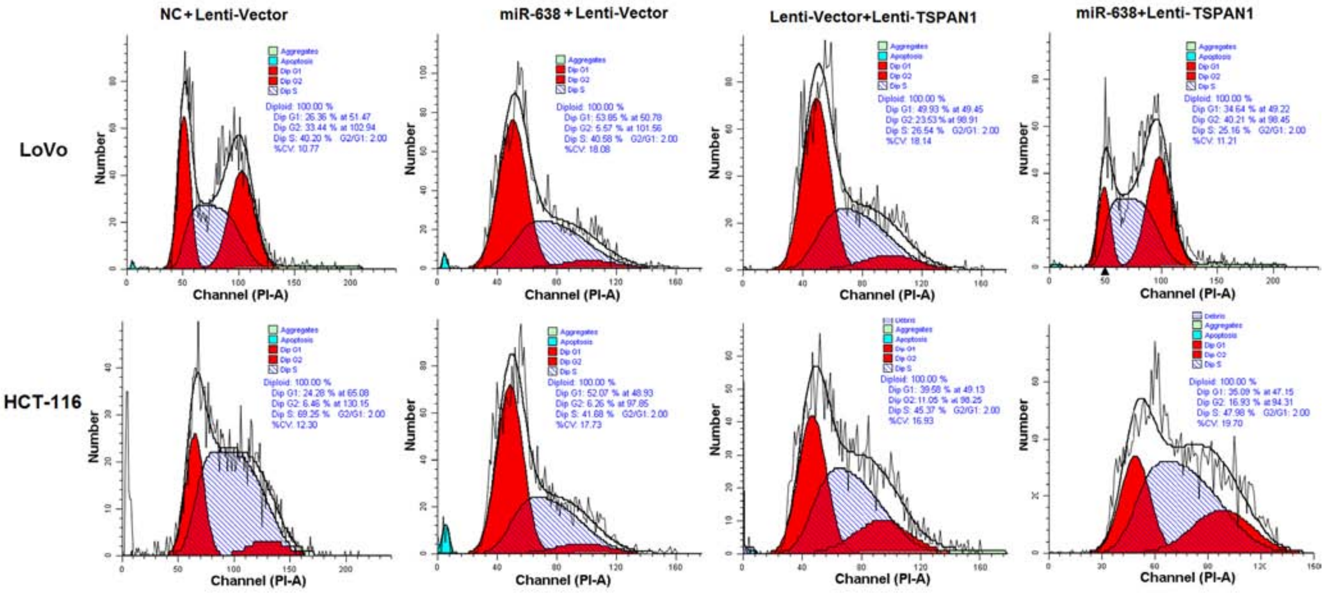

Supplementary Figure S4: Representative graphs of cell cycle analysis. Cell cycle analyses were performed in LoVo and HCT-116 cells transfected with miR-638, TSPAN1(ORF without 3’UTR) or vector control.

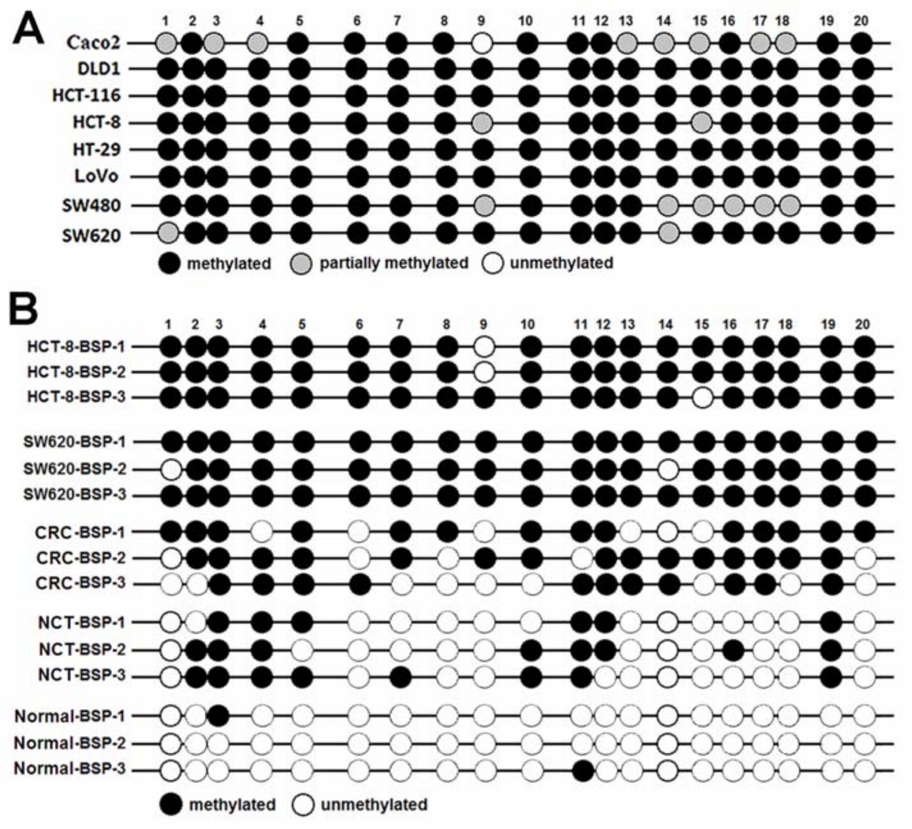

Supplementary Figure S5: Representative results of bisulfite-sequencing PCR (BSP). (A) Schematic representation of the methylation status of the CpG island (including 20 CpG sites) as detected in the indicated CRC cell lines. **(B)** Three positive clones were selected from T-A cloning of BSP products from CRC cells (HCT-8, SW480) or clinical tissue samples (CRC, NCT or normal colonic epithelia) and subjected to sequencing.

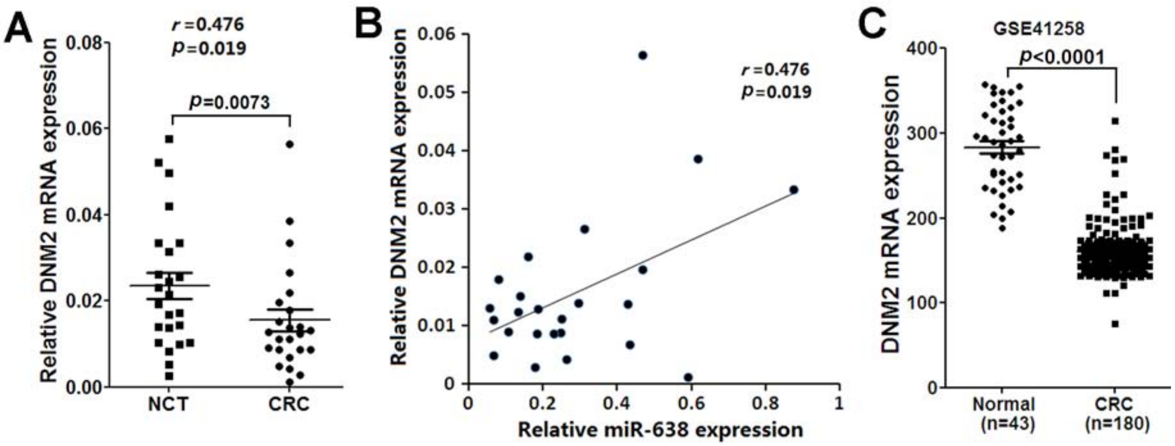

Supplementary Figure S6: DNM2 mRNA expression is downregulated in CRC. **(A)** DNM2 mRNA expression was detected by qRT-PCR in 24 paired CRC and adjacent noncancerous tissues (NCTs). **(B)** Relative DNM2 mRNA expression was positively correlated with miR-638 expression in CRC tissues. **(C)** DNM2 mRNA expression was downregulated in CRC compared with NCT in a test cohort. The data of DNM2 mRNA expression was obtained from a published CRC microarray data set (GSE41258).

Supplementary Table S2. Patients' information

Characteristics	Colorectal cancer* (n=146)	Colorectal cancer# (n=113)	Colorectal cancer$ (n=103)
**Gender**			
Male	83	68	61
Female	63	45	42
**Age at diagnosis**			
≥58	76	65	60
<58	70	48	43
**TNM stage**			
I	20	17	16
II	45	32	29
III	61	50	45
IV	20	14	13
**Nodal status**			
Positive	79	62	56
Negative	67	51	47
**Tumor size (cm)**			
<5	87	66	60
≥5	59	47	43
**Tumor location**			
Rectum	62	50	42
Distal colon	38	30	28
Proximal colon	46	33	33
**Grading**			
G1	6	4	4
G2	114	88	79
G3	26	21	20

*These cases were subjected to the detection of TSPAN1 protein.
#These cases were subjected to the detection of miR-638 expression.
$Both the miR-638 and TSPAN1 expression data were available in 103 cases.
